# Antidiabetic Activity, Molecular Docking, and ADMET Properties of Compounds Isolated from Bioactive Ethyl Acetate Fraction of *Ficus lutea* Leaf Extract

**DOI:** 10.3390/molecules28237717

**Published:** 2023-11-22

**Authors:** Oyinlola O. Olaokun, Muhammad S. Zubair

**Affiliations:** 1 Department of Biology and Environmental Science, School of Science and Technology, Sefako Makgatho Health Science University, Molotlegi Street, Ga-Rankuwa, Pretoria 0204, South Africa; 2 Natural Product Research Group, Department of Pharmacy, Faculty of Science, Tadulako University, Palu-Central Sulawesi 94118, Indonesia; sulaimanzubair@untad.ac.id

**Keywords:** *Ficus lutea*, antidiabetic, molecular docking, ADMET, phytochemical

## Abstract

Diabetes contributes to the rising global death rate. Despite scientific advancements in understanding and managing diabetes, no single therapeutic agent has been identified to effectively treat and prevent its progression. Consequently, the exploration for new antidiabetic therapeutics continues. This study aimed to investigate the antidiabetic bioactive ethyl acetate fraction of *F. lutea* at the molecular level to understand the molecular interactions and ligand-protein binding. To do this, the fraction underwent column chromatography fractionation to yield five compounds: lupeol, stigmasterol, α-amyrin acetate, epicatechin, and epiafzelechin. These compounds were evaluated in vitro through α-glucosidase inhibition and glucose utilization assays in C2C12 muscle and H-4-11-E liver cells using standard methods. In silico analysis was conducted using molecular docking and ADMET studies. Epicatechin exhibited the most potent α-glucosidase inhibition (IC_50_ = 5.72 ± 2.7 µg/mL), while epiafzelechin stimulated superior glucose utilization in C2C12 muscle cells (33.35 ± 1.8%) and H-4-11-E liver cells (46.7 ± 1.2%) at a concentration of 250 µg/mL. The binding energies of the isolated compounds for glycogen phosphorylase (1NOI) and α-amylase (1OSE) were stronger (<−8.1) than those of the positive controls. Overall, all tested compounds exhibited characteristics indicative of their potential as antidiabetic agents; however, toxicity profiling predicted epiafzelechin and epicatechin as better alternatives. The ethyl acetate fraction and its compounds, particularly epiafzelechin, showed promise as antidiabetic agents. However, further comprehensive studies are necessary to validate these findings.

## 1. Introduction

Diabetes, a non-communicable disease (NCD), contributes significantly to the rising global death rate [1]. By 2030, it is projected to be the leading cause of death in Africa [2]. This condition is characterized by persistently high blood glucose levels due to insufficient insulin secretion, cellular insensitivity to insulin, or both, and is often linked to pancreatic β-cell failure [3]. With alarming mortality rates, diabetes has emerged as a major underlying cause of death worldwide [4,5]. The International Diabetes Federation (IDF) reported a surge in global diabetes prevalence from 151 million adults in 2000 to 451 million in 2017, with a predicted increase to 693 million by 2045, especially in lower to middle-income countries, if no action is taken [6].

While several conventional medications exist for diabetes management, their affordability for low-income individuals and issues related to prolonged use, such as adverse effects and poor patient compliance, remain be to challenges [7]. These therapies often fail to halt disease progression, leading to eventual insulin dependence [8]. The primary goal of treatment is to achieve normal glycemia levels to prevent complications. However, global efforts to find a successful treatment have not yet been realized. Consequently, the exploration of new antidiabetic therapeutics continues, with medicinal plants being considered a viable alternative. Medicinal plants are rich sources of phytochemicals, possessing the ability to alleviate various ailments and diseases. Their pharmacological properties stem from secondary metabolites, which are natural sources of bioactive compounds. Since diabetes affects multiple pathways in different tissues, these pathways serve as targets for drug development in monotherapy [9]. Extracts containing bioactive secondary metabolites can be utilized in polytherapy to target multiple pathways, enhancing treatment outcomes [10]. While drug discovery typically focuses on isolating single lead compounds, identifying and characterizing bioactive phytochemical compounds within extracts that collectively modulate multiple pathways to ameliorate disease progression is crucial. 

Traditionally, plants belonging to the *Ficus* genus, including *Ficus lutea*, have been used for their medicinal properties, such as antidiabetic, anthelmintic, hypotensive, mild laxative, antirheumatic, digestive, and anti-dysentery remedies [11,12]. These properties are attributed to chemical constituents like triterpenes, sterols, polyphenols, flavonoids, coumarins, alkaloids, and other metabolites [13]. *F. lutea*, in particular, has been identified for its potential in managing diabetes [13]. 

*Ficus lutea* Vahl, a member of the Moraceae family, is commonly referred to as the African wild fig or yellow leaf rock fig. This distinctive fig tree species is native to various regions of Africa and is characterized by a spreading canopy that can reach heights of 20 m or more, with an extensive root system enabling its growth on rocky surfaces, both solitary and in clusters. The bark of the tree starts grayish-brown, appearing smooth but gradually developing slight fissures with age, eventually becoming rougher and more textured as the tree matures. The leaves are large, simple, and arranged alternately along the branches, featuring prominent veins, an oval to elliptical shape, and a pointed tip. While they are typically dark green, they can also exhibit a yellow tint. The fruit of *F. lutea*, known as syconium, begins as green and ripens to a yellow shade. Inside the syconium, small flowers and seeds are enclosed [14].

Studies have highlighted the antidiabetic potential of *F. lutea* leaf crude acetone extract. This potential was demonstrated through the inhibition of digestive enzymes (α-amylase and α-glucosidase), stimulation of glucose utilization in muscle and adipocytes, and the insulin-releasing action of insulinoma cells [13,15]. In particular, the antidiabetic potential of the *F. lutea* leaf crude acetone extract was found to be stronger in the ethyl acetate fraction, which exhibited potent in vitro antidiabetic activity [16]. Furthermore, in vivo studies demonstrated the potential of the ethyl acetate fraction of *F. lutea* extract to ameliorate hyperglycemia and obesity in an obese mouse model to some extent [16]. The possible additive or synergistic therapeutic effects of the bioactive phytochemicals within the ethyl acetate fraction may likely be responsible for the perceived effects and may be beneficial for diabetes management [10].

To further explore the antidiabetic potential of the ethyl acetate fraction, a procedure was undertaken that fractionated the bioactive ethyl acetate fraction using silica gel column chromatography. This process led to the isolation of five compounds, which were elucidated using nuclear magnetic resonance (NMR) [8]. While a previous study had delved into the antidiabetic potential of *F. lutea* extract and its fractions [16], there remained a gap in understanding the molecular-level interactions, including ligand-protein binding and potential metabolism. To address this, experimentation with isolated compounds was initiated. Consequently, this study had two main objectives: first, to investigate the in vitro antidiabetic potential of the five compounds from the bioactive ethyl acetate fraction of *F. lutea*; and second, to predict, through in silico analysis, the binding interactions via molecular docking as well as the ADMET pharmacokinetic characteristics.

## 2. Results

### 2.1. Phytochemical Screening and Compound Identification

**Figure 1:**
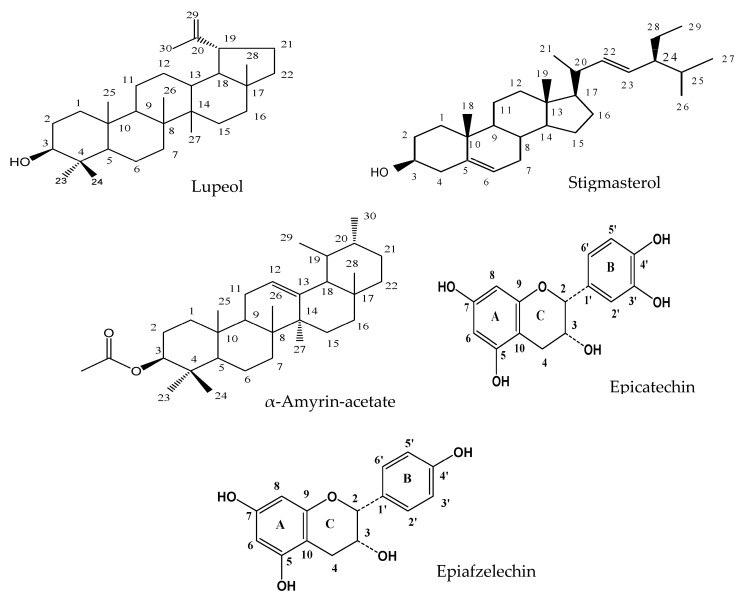
Elucidated structures of compounds of the ethyl acetate fraction of *F. lutea* leaf acetone extract.

The structures of the compounds isolated from the antidiabetic bioactive ethyl acetate fraction of *F. lutea* elucidated by NMR are presented in Figure 1. 

The result of the qualitative phytochemical screening of the isolated compounds revealed the presence of terpenoids, steroids, and flavonoids in the ethyl acetate fraction (Table 1).

Compound **1**, obtained as a white powder, gave a pink coloration in the Liebermann-Buchard’s test, which is characteristic of triterpenes. The ^1^H and ^13^C NMR spectra (Figures S1 and S2 in Supplementary Data) exhibited signals at δ 4.68 (brs, H-29a), 4.47 (brs, H-29b), 3.18 (m, H-3), 109.4 (C-29), and 79.1 ppm (C-3), assignable, respectively, to protons and carbons of methylene and oxymethine groups at positions 29 and 3 of a lup-20(29)-en-3-ol class of triterpenes [17]. A signal for carbon, C-20, of the lupeol structure was observed on the ^13^C NMR spectrum at δ 148.2 ppm, while seven singlet signals relevant for angular methyl groups appeared on the ^1^H NMR spectrum between 0.8 and 1.7 ppm. This compound has been previously isolated from the same species [18]. 

Compound **2**, obtained as a white powder, gave a green-blue coloration in the Liebermann-Buchard’s test, which is characteristic of sterols. The presence of 30 carbons on the ^13^C NMR spectrum (Figure S3 in Supplementary Data), including signals due to six angular methyl groups, indicated that this compound was a phytosterol. The ^13^C NMR spectrum displayed signals at δ 140.7, 138.3, 129.2, 121.7, and 71.8 ppm, corresponding, respectively, to carbons C-5, C-22, C-23, C-6, and C-3 of the stigmasterol structure. The ^1^H NMR spectrum (Figure S4 in Supplementary Data) exhibited signals at δ 5.32 (brd, 2.2; 3.0 Hz, 1H), 5.17 (dd, 8.5 Hz, 1H), 5.00 (dd, 8.5 Hz, 1H), and 3.50 (m, 1H), corresponding to protons H-6, H-22, H-23, and H-3, respectively. Signals due to methyl groups were observed on the ^1^H NMR between 0.90 and 0.60 ppm and agreed with those of previously reported stigmasterol [19].

Compound **3**, obtained as an oil, gave a pink coloration in the Liebermann-Buchard’s test, which is characteristic of triterpenes. The ^13^C NMR spectrum (Figure S5 in Supplementary Data) exhibited 30 carbons with 4 characteristic downfields displayed at δ 170.9, 139.7, 124.4, and 81.0, assignable to a triterpene skeleton with one carbonyl acetate (CH3CO), one ethylenic double bond (C=CH), and one oxymethine (HCOH) group. The ^1^H NMR spectrum (Figure S6 in Supplementary Data) exhibited characteristic signals at δ 5.12 (t, 3.7 Hz, 1H), 4.49 (m, 1H), and 2.02 (s, 3H) due to protons at positions C-12, C-3, and the acetyl group from α-amyrin acetate [17]. All of the data above were similar to those published for α-amyrin acetate previously isolated from the same species [18]. 

Compound **4**, obtained as a yellowish powder, gave a positive result in the Shinoda test characteristic of flavonoids. The ^1^H NMR spectra (Figure S7 in Supplementary Data) showed singlets at δ 8.10, 7.94, 7.79, and 7.74 ppm, assignable to four phenolic protons. The ^1^H NMR spectrum also exhibited a broad singlet, one multiplet, and two doublets of doublet signals at δ 4.87 (brs, 1H), 4.20 (m, 1H), 2.85 (dd, 4.6, 16.5 Hz, 1H), and 2.72 (dd, 3.3, 16.7 Hz, 1H), attributable to protons H-2, H-3, H-4b, and H-4a, respectively. The ^1^H NMR spectrum showed two sets of aromatic protons: the first one at δ 6.01 (d, 2.3 Hz, H-6) and 5.91 (d, 2.3 Hz, H-8), corresponding to the A ring, and the second one at δ 7.04 (d, 2.0 Hz, H-2’), 6.88 (d, 8.4 Hz, H-5’), and 6.83 ppm (dd, 2.0, 8.4 Hz, H-6’), attributable to B ring protons. The ^13^C NMR spectrum (Figure S8 in Supplementary Data) exhibited the characteristic flavan-3-ol signals at δ 79.4, 66.9, and 28.9 ppm, corresponding to C-2 (OCH), C-3 (COH), and C-4 (CH2), respectively. The ^13^C NMR spectrum exhibited 12 aromatic carbons at δ 145.2, 145.3, 157.1, 157.5, and 157.6 ppm, attributable, respectively, to five oxygenated carbons C-3’, C-4’, C-5, C-7, and C-9, and at δ 132.2, 115.2, 115.4, 119.3, 96.1, 96.0, and 99.8 corresponding to carbons C-1’, C-2’, C-5’, C-6’, C-6, C-8, and C-10, respectively. All the data for this compound agreed with those reported for epicatechin previously isolated from lychee fruit pericarp tissues [20].

Compound **5**, obtained as a yellowish powder, displayed properties typical of a flavonoid, with positive results in the Shinoda test for flavonoids, and has been previously characterized as epiafzelechin by authors [15].

### 2.2. Inhibition of α-Glucosidase Activity and Glucose Utilization Enhanced by the Compounds

The compounds isolated from the antidiabetic bioactive ethyl acetate fraction were evaluated for their inhibitory effect on α-glucosidase activity (Table 2). The potency of all the isolated compounds against the activity of α-glucosidase was weak compared to that of the positive control, acarbose, which gave an IC_50_ value of 1.52 ± 0.05 µg/mL. Among the compounds, the high inhibitory potency of epicatechin (5.72 ± 2.7 µg/mL) was not significantly different (*p* ≤ 0.05) from that of epiafzelechin (7.64 ± 37.5 µg/mL), while the weakest potency was recorded for lupeol (IC_50_ > 1000 µg/mL).

**Figure 2:**
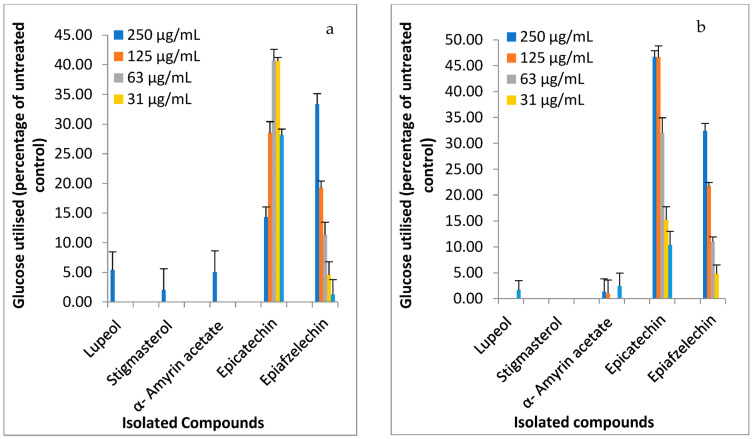
Glucose utilization in (**a**) C2C12 muscle cells and (**b**) H-4-11-E rat liver cells (expressed as a percentage of untreated control cells ± standard error of the mean, n = 9) exposed to compounds isolated from the ethyl acetate fraction of *F. lutea* leaf acetone extract.

The C2C12 muscle cells treated with the isolated compounds exhibited glucose utilization at a concentration of 250 µg/mL (Figure 2a). Specifically, epiafzelechin stimulated a superior increase (33.35 ± 1.8%) in glucose utilization at this concentration. Both epiafzelechin and epicatechin were effective in promoting glucose utilization in C2C12 muscle cells compared to other compounds. Epiafzelechin also exhibited a concentration-dependent enhancement in glucose utilization in C2C12 cells compared with epicatechin. Furthermore, H-4-11-E liver cells treated with isolated compounds at 250 µg/mL, except for stigmasterol, displayed glucose utilization activity, as shown in Figure 2b. The glucose utilization in H-4-11-E liver cells was concentration-dependent, with epiafzelechin and epicatechin enhancing glucose utilization of 46.7 ± 1.2% and 32.4 ± 1.5%, respectively, at 250 µg/mL. 

### 2.3. Molecular Docking of Isolated Compounds against Glucose-Metabolizing Receptors

The docking study of compounds isolated from the ethyl acetate fraction resulted in 125 docking conformations. A lower binding energy, or a more negative free energy of binding, generally signifies greater stability and binding affinity between the compound and the receptors [21]. Out of these conformations, eight poses exhibited the best free energy of binding (kcal/mol), favoring interactions with receptors 1NOI, 3G9E, 2P8S, 5EQG, 4RCH, 5T19, 1OSE, and 2QMJ (Table 3). All five compounds were predicted to possess strong binding affinity for these receptors. Particularly, the binding energies of these compounds for glycogen phosphorylase (1NOI) and α-amylase (1OSE) were stronger (<−8.1 kcal/mol) than those of the positive controls. Stigmasterol, which exhibited the lowest binding energy, displayed an affinity for five out of the eight receptors (3G9E, 5EQG, 4RCH, 1OSE, and 2QMJ). Following closely was lupeol, a terpenoid demonstrating a binding affinity for three of the eight receptors (1NOI, 2P8S, and 5T19). 

**Figure 3:**
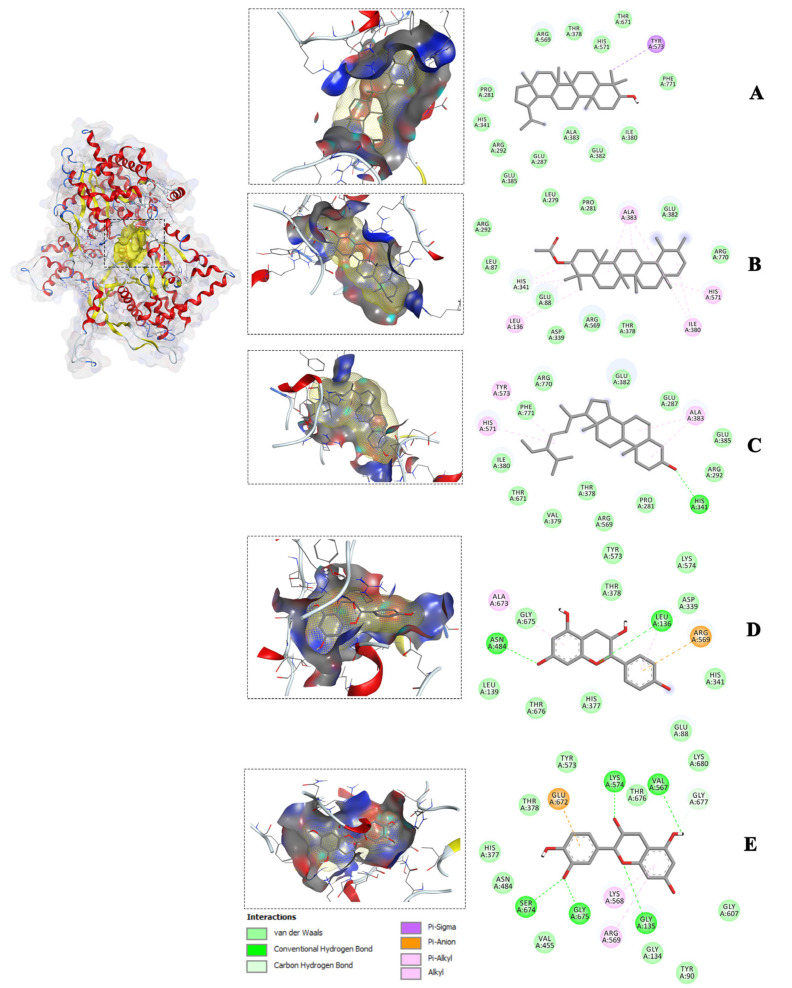
Molecular interactions of the isolated compounds ((**A**) = Lupeol, (**B**) = a-Amyrin-acetate, (**C**) = Stigmasterol, (**D**) = Epiafzelechin, and (**E**) =Epicatechin) from the ethyl acetate fraction with the lowest binding affinity against the Glycogen phosphorylase receptor (PDB ID: 1NOI).

**Figure 4:**
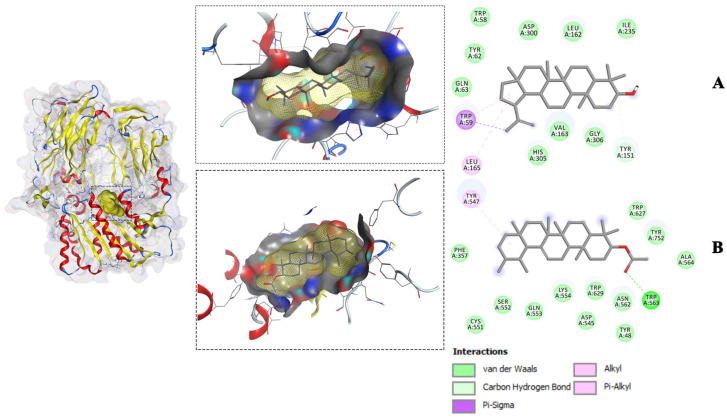
Molecular interactions of the isolated compounds ((**A**) = Lupeol, (**B**) = a-Amyrin-acetate) from ethyl acetate fraction with the lowest binding affinity against the Dipeptidyl peptidase 4 (DPP-IV) receptor (PDB ID: 2P8S).

**Figure 5:**
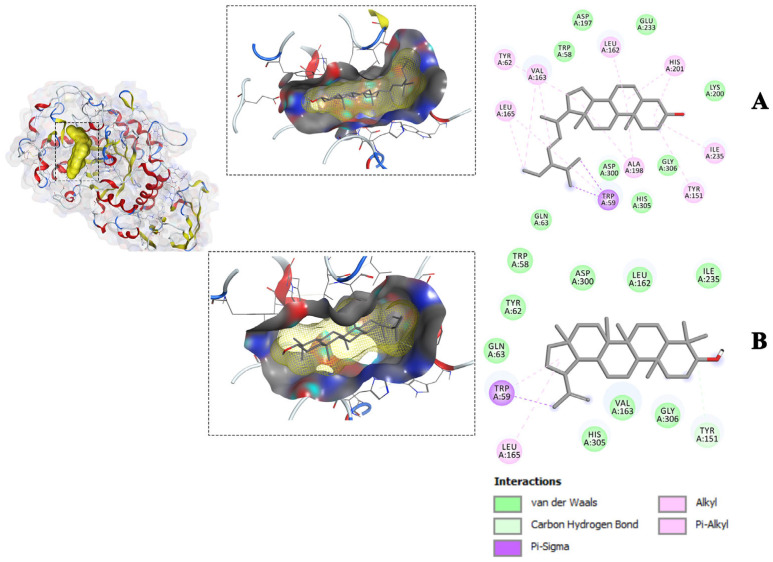
Molecular interactions of the isolated compounds ((**A**) = Stigmasterol, (**B**) =Lupeol) from the ethyl acetate fraction with the lowest binding affinity against the D α-Amylase receptor (PDB ID: 1OSE).

To gain further insight into the interactions between the compounds and the amino acid residues of the receptors, the study focused on the most favorable compound-receptor poses, which exhibited lower binding energy compared to the native ligand poses. These selected binding poses were identified for glycogen phosphorylase (1NOI), dipeptidyl peptidase (2P8S), and α-amylase (1OSE), and are visualized in Figure 3, Figure 4 and Figure 5. In both the 3D and 2D visualizations, these binding poses revealed various interaction types, including hydrogen bonds, hydrophobic interactions, van der Waals forces, and the specific amino acid residues involved in the binding interactions (Figure 3, Figure 4 and Figure 5). It is noteworthy that the amino acid residues involved in the interactions and the types of interactions varied among the different compounds. For instance, stigmasterol interacted with four amino acids of the 1NOI receptor and formed a hydrogen bond, while lupeol interacted with only one amino acid and α-amyrin acetate with six amino acids but without hydrogen bonding. A detailed analysis of individual amino acids revealed that α-amyrin acetate interacted with three amino acids (HIS571, ALA383, and HIS341), while lupeol interacted with one amino acid (TYR573) out of the four amino acids that stigmasterol interacted with (HIS571, TYR573, ALA383, and HIS341) within the binding site of the glycogen phosphorylase receptor (1NOI). Moreover, stigmasterol formed a hydrogen bond with the amino acid HIS341 of 1NOI, whereas α-amyrin acetate interacted with the same amino acid through van der Waals interactions. Additionally, lupeol and stigmasterol both interacted with the same three amino acids—LEU165, TRP59, and TYR151—at the binding site of α-amylase (1OSE). These two compounds interacted with amino acids LEU165 and TRP59 through π-alkyl and π-sigma interactions, respectively. These intricate interactions demonstrated the diverse molecular mechanisms underlying the compounds’ binding affinity with the different receptors.

### 2.4. Drug-Likeness and ADMET Properties

The drug-likeness of the five isolated compounds was evaluated using the physicochemical properties outlined in Table 4. While all parameters indicated that epiafzelechin and epicatechin are within acceptable ranges, lupeol, stigmasterol, and α-amyrin acetate violated the rule due to higher Log *p*-values (>5). However, according to Lipinski’s rule [22], which defines criteria for potential drug candidates, all five compounds exhibited characteristics indicative of their potential as drug candidates.

The predicted ADME (Absorption, Distribution, Metabolism, and Excretion) pharmacokinetic properties of the isolated compounds are outlined in Table 5, providing insights into their potential therapeutic applicability. Regarding absorption kinetics, both water solubility and gastrointestinal (GI) absorption were superior for the flavonoids–epicatechin and epiafzelechin compared to the other compounds. With the exception of epicatechin, all isolated compounds exhibited high Caco-2 membrane permeability (log Papp value > 0.9 cm/s), indicating their potential for absorption within the human body. The skin permeability values for all compounds are below the normal threshold (log kp ≥ −2.5 cm/s), suggesting limited absorptive capabilities through the skin. Concerning their interaction with P-glycoprotein (P-gp), a crucial cellular efflux transporter, epicatechin, and epiafzelechin were identified as substrates for P-gp, potentially impacting their absorption and distribution within the body. Conversely, lupeol, stigmasterol, and α-amyrin acetate were identified as P-gp I/II inhibitors, potentially affecting their bioavailability and systemic distribution.

The compounds’ potential to permeate and distribute across various physiological barriers was investigated (Table 5). All compounds displayed steady-state volume of distribution (VDss) values exceeding the lower limit (>−0.15), indicating their widespread distribution within the body. Epicatechin and epiafzelechin exhibited higher VDss values (>0.45) compared to the other three compounds, suggesting their extensive tissue distribution rather than in the plasma. A higher unbound fraction was equally recorded for epicatechin and epiafzelechin, indicating better cell membrane permeation. However, lupeol, stigmasterol, and α-amyrin-acetate recorded a zero unbound fraction, signifying their strong binding affinity to proteins. Another aspect of lupeol, stigmasterol, and α-amyrin-acetate is their ability to traverse the blood-brain barrier (BBB), as evidenced by log BB values > 0.3, and their distribution into the central nervous system (CNS), as evidenced by log PS values > −2, which may have a potential neurological effect.

Metabolism prediction was conducted by assessing the interaction of the isolated compounds with cytochrome P450 isoforms, considering their roles as substrates or inhibitors (Table 5). None of the compounds were found to inhibit cytochrome P450 isoenzymes. However, it was identified that lupeol, stigmasterol, and α-amyrin acetate serve as substrates for CYP3A4 isoenzymes, indicating possible modulation of metabolic pathways mediated by CYP3A4.

Regarding excretion kinetics, none of the compounds were predicted as substrates for the renal organic cation transporter 2 (OCT2). Stigmasterol exhibited the highest total clearance score (0.618 log mL/min/kg), indicating its efficient elimination from the body, while α-amyrin acetate displayed the lowest score (0.025 log mL/min/kg), suggesting a comparatively slower clearance rate.

The toxicity assessments outlined in Table 6 provide insights into the safety profiles of the isolated compounds. Epicatechin was classified as class 6 (LD50 > 5000), indicating its relatively non-toxic nature. Similarly, both epiafzelechin and α-amyrin-acetate were classified as class 5 (2000 < LD50 ≤ 5000), highlighting their low toxicity. In contrast, lupeol and stigmasterol, categorized into class 4 (300 < LD50 ≤ 2000), indicate a higher toxicity level. The acute rat oral toxicity (LD_50_) predictions for all isolated compounds aligned closely, averaging at 2.43 ± 0.13 mol/kg. This consistency highlights the uniformity of their immediate toxic effects. However, concerning chronic rat oral toxicity, lupeol, and stigmasterol exhibited higher toxic values (0.881 log mg/kg/day) compared to the other compounds, suggesting potential long-term health risks associated with their usage. The identification of lupeol, stigmasterol, and α-amyrin-acetate as hERG II inhibitors raises concerns about their potential impact on cardiac health. Moreover, the predicted immunotoxicity of these three compounds indicates their impact on the immune system. The absence of hepatotoxicity, skin sensitization, mutagenicity in AMES tests, and the lack of identification as hERG I inhibitors are positive findings.

The possible ecological impact of the compounds was assessed using in silico environmental toxicology models of *Tetrahymena pyriformis* and minnow larvae (Table 6). The compounds exhibited a predicted toxic dose against *T. pyriformis* ranging from 0.316 to 0.519 µg/L, with lupeol displaying the highest toxicity. When evaluating their effects on minnow larvae, the negative toxicity scores of lupeol, stigmasterol, and α-amyrin-acetate were more potent than those of the flavonoid compounds. This disparity implies varying levels of environmental impact among the compounds. 

## 3. Discussion

The exploration of bioactive compounds in *F. lutea* has uncovered promising candidates for potential antidiabetic agents. Among the isolated compounds, epicatechin, epiafzelechin, and stigmasterol demonstrated inhibitory effects on α-glucosidase activity. α-Glucosidase is an enzyme in the small intestine crucial for glucose metabolism. It is involved in the breakdown of complex carbohydrates into simpler sugars like glucose, facilitating absorption and raising blood sugar levels. As α-Glucosidase inhibitors, epicatechin, epiafzelechin, and stigmasterol can slow down the enzyme’s action, reducing carbohydrate conversion into glucose, lowering post-prandial blood sugar levels, and aiding diabetes management. Furthermore, epicatechin and epiafzelechin exhibited enhanced glucose utilization in both C2C12 muscle cells and H-4-II-E liver cells. The skeletal muscle plays a crucial role in maintaining blood glucose homeostasis by serving as the primary site for glucose uptake, accounting for about 75% of glucose disposal after a meal. Additionally, the liver, a key organ in glycemic regulation, stores energy as glycogen and triglycerides. The ability of these two compounds to enhance glucose utilization in cells suggests their potential as antidiabetic agents. These findings implied that the antidiabetic potential of the ethyl acetate fraction may probably be due to the synergistic action of these bioactive compounds and/or other terpenoids, steroids, and flavonoids contributing to the plant’s antidiabetic properties. Following this, molecular docking studies were employed to elucidate the binding mechanisms of these compounds with receptors involved in antidiabetic activity, utilizing an in-silico approach.

Molecular docking studies are frequently employed in drug design to predict interactions between ligands and proteins. This is achieved by calculating the binding affinity and visualizing the amino acid interactions contributing to it. Docking enables the prediction of antidiabetic activity by assessing the binding affinity of isolated compounds for proteins involved in glucose metabolism. Antidiabetic therapies are typically developed to target various mechanisms of glucose metabolism, involving multiple pathways [23]. In this study, molecular docking was performed against twelve receptors (α-amylase, α-glucosidase, PPAR-γ, IGF1R, DPP-IV, GLUT1, SUR, GP, IR, GK, PTP1B, and SGT2) identified in the literature as playing important roles in glucose metabolism to determine their efficacy [23]. A total of 125 docking analyses were conducted for the five isolated compounds against these twenty-five receptors. The binding conformation of the compounds within the active site of the receptors was assessed based on the scoring function and predicting the strength of the compound-receptor interaction. Forty molecular docking interactions were selected because they had the best (lower scores) free energy of binding (ΔG kcal/mol). All the compounds interacted with the receptors to varying degrees. The binding affinities, evaluated through scoring functions, identified stigmasterol as the most promising compound, demonstrating a strong affinity for a broad spectrum of receptors, followed closely by lupeol. These compounds exhibited superior binding to multiple receptors, suggesting their potential as candidates for antidiabetic drug development. However, these in silico results contrasted with the findings from the in vitro α-glucosidase inhibitory and glucose utilization assays in cells, where epicatechin and epiafzelechin demonstrated favorable activity. Notably, epicatechin, epiafzelechin, and the conventional antidiabetic drugs (i.e., the positive controls) did not exhibit superior binding affinities for the protein receptors. This observation might indicate that epicatechin, epiafzelechin, and the positive controls interacted with the protein receptors in a similar manner, suggesting a limitation in the results of the molecular docking analysis.

In drug development, effective binding to the target is not only essential but also ensures oral bioavailability [24] and drug-likeness properties [25]. In this regard, examining the physicochemical properties of the compounds is crucial for drug development [26]. The adherence to Lipinski’s rule [22] by epiafzelechin and epicatechin positions them as active drug candidates. Lipinski’s rule of five is a set of criteria used to evaluate the drug-likeness of small molecules, which includes molecular weight ≤ 500 g/mol, Log P (octanol-water partition coefficient) ≤ 5, hydrogen bond acceptors ≤ 10, hydrogen donors ≤ 5, and topological polar surface area (≤140), with only one violation permitted (Table 4). Compounds that violate more than one of Lipinski’s rules are unlikely to be active drug candidates [27]. Compounds with high log *p*-values like lupeol, stigmasterol, and α-amyrin-acetate may pose challenges in reaching therapeutic targets due to their lipophilicity, potentially limiting their efficacy [28]. Favorable bioavailability scores (0.55) predict good suitability for oral drug applications [29,30], implying that a smaller quantity of the compound is required to achieve the expected therapeutic outcome, reducing the risk of side effects and toxicity. Also, compounds with molecular weights > 250 g/mol ≤ 350 g/mol and lower hydrophobicity than those specified by Lipinski’s rule are positioned as lead-like candidates [31]. In this regard, epiafzelechin and epicatechin exhibit lead-like characteristics [30,32], indicating their potential as candidates in the drug discovery process. Consequently, lead-likeness, adherence to Lipinski’s rule, and favorable oral bioavailability are suggested as the characteristics of compounds pivotal in drug discovery.

To develop an effective therapeutic agent, it is crucial a drug candidate reaches its target location in sufficient concentration, inducing the desired pharmacologic effect while minimizing side effects. Predictive ADMET analyses offer valuable insights into how compounds behave in the human body, revealing their interactions with proteins, distribution patterns, and metabolism pathways [33]. Epicatechin and epiafzelechin exhibited excellent gastrointestinal absorption, indicating their potential as orally administered agents crucial for antidiabetic therapy [24,34]. The intestine, with its large surface area, serves as the primary site for drug absorption when administered orally [35,36]. P-glycoprotein (P-gp), an ATP-binding cassette efflux transporter, acts as a biological barrier [37], limiting the absorption of drugs and natural compounds from the gut by pumping xenobiotics out of cells to protect against toxic substances [37]. While substrates of P-gp are easily pumped out of cells, inhibitors of P-gp I/II can enhance the absorption and distribution of chemicals, leading to therapeutic or adverse effects. Epicatechin and epiafzelechin, functioning as P-glycoprotein substrates, might have their absorption affected. In contrast, lupeol, stigmasterol, and α-amyrin acetate, acting as P-glycoprotein inhibitors, exhibited low water solubility and poor intestinal absorption. These findings offer valuable insights for designing effective therapeutic regimens [38]. Depending on the mode and type of P-gpI/II inhibition, lupeol, stigmasterol, and α-amyrin acetate could potentially facilitate the absorption of flavonoid compounds into cells when the ethyl acetate fraction is administered. Among the compounds, only epicatechin, with a recorded negative value, is predicted to have moderate Caco-2 absorption. Caco-2, a human colon epithelial cancer cell line, is modeled to simulate the properties of the human small intestine. It expresses enterocytes, transporters, cytochrome P450 enzymes, and efflux proteins [37,39], making it a gold standard for predicting in vitro human intestinal chemical permeability. All the isolated compounds show poor absorption through the skin, a transdermal route irrelevant in the administration of antidiabetic therapeutics.

The compounds’ potential for distribution within the body was evaluated using parameters such as steady-state volume of distribution (VDss) and compound-protein binding. Compounds with higher VDss values (VDss > 0.45), such as epicatechin and epiafzelechin, tend to distribute more in tissues than in plasma [36], potentially enhancing therapeutic effects. The ability of lupeol, stigmasterol, and α-amyrin-acetate to bind to proteins in the blood can impact efficacy because the unbound fraction of a compound is the portion that can exert therapeutic effects [40], as it is available to permeate through the cell membrane [36,37]. Strong protein binding may also lead to prolonged therapeutic effects. Furthermore, compounds like lupeol, stigmasterol, and α-amyrin-acetate, which can cross the blood-brain barrier [33], raise concerns about potential neurological effects that may induce positive, negative, or toxic effects.

After absorption, the chemical compounds undergo metabolism in the liver, where cytochrome P450 isoenzymes (CYP1A2, CYP3A4, CYP2C9, CYP2C19, and CYP2D6) play a crucial role in drug safety, persistence, and bioactivation [38]. None of the isolated compounds are predicted to be inhibitors of the CYP450 enzymes. This is favorable because inhibitors can block the substrate’s binding site, modify enzymatic activity, slow down metabolism, and lead to the accumulation of the substrate in the body [40]. Lupeol, stigmasterol, and α-amyrin-acetate, however, are substrates of the CYP3A4 isoenzyme. As substrates, these compounds may be transformed into metabolites that could either be inactive for clearance or activated to produce beneficial or undesirable effects.

The amount of chemical compound removed from plasma in the vascular compartment per unit time is known as clearance [37]. The total clearance score encompasses all hepatic and renal clearances of the compound excreted via the kidneys [37,41]. Stigmasterol exhibited the highest clearance score, indicating rapid elimination from the body. This suggests a shorter half-life and necessitates more frequent dosing to maintain therapeutic levels in the bloodstream. On the other hand, α-amyrin-acetate recorded a low clearance score, suggesting the compound is eliminated from the body at a relatively slow rate. This slower clearance results in a longer half-life, potentially requiring less frequent dosing to maintain therapeutic levels. Additionally, the renal uptake transporter Organic Cation Transporter 2 (OCT2) plays a vital role in drug disposition and renal clearance. None of the five compounds are substrates for OCT2, which is essential for the excretion of cationic molecules.

The toxicity below the detectable limit is a crucial factor when selecting a compound as a therapeutic candidate [42]. The study evaluated the toxicity of potential therapeutic compounds using various parameters. The Maximum Tolerable Dose (MTD), estimating the highest dose at which a potential drug exhibits pharmacological activity without toxicity [33,36], predicted that all the compounds exhibited low MTD values (≤0.477 log mg/kg/day). Lupeol, stigmasterol, and α-amyrin-acetate had particularly unfavorable MTD values, specifying tolerated doses. The possibility of compounds causing adverse effects from repeated exposure over a long period of time in an oral rat chronic toxicity test [43] was estimated. In the rat tests, lupeol and stigmasterol were predicted to cause adverse effects at low doses, known as the Lowest Observed Adverse Effect Level (LOAEL) [33,36]. None of the compounds was predicted to exert organ toxicity or mutagenicity. However, lupeol, stigmasterol, and α-amyrin-acetate were found to inhibit hERG II, indicating potential cardiotoxicity. The hERG (human ether-à-go-go-related gene) inhibition could lead to cardiac arrhythmias [33]. This is because hERG encodes for a potassium channel with a fundamental role in cardiac action, the potential repolarization inhibition of which may lead to QT interval prolongation, ventricular tachycardia, and even death [33]. These compounds were also predicted to exhibit immunotoxicity. Environmental impact predictions indicated moderate toxicity for most compounds against *T. pyriformis*, with lupeol being relatively more toxic. The concentration that inhibited 50% growth (pIG_50_) of *T. pyriformis* with pIG_50_ = −0.5 log µg/L is considered a toxic concentration [44]. For minnow larvae, lupeol, stigmasterol, and α-amyrin-acetate were highly toxic. The concentration that caused the death of 50% of the Flathead minnows is considered highly toxic if LC_50_ values are below 0.5 mM (log LC50 < 0.3) [39]. The flavonoid compounds seemed more environmentally favorable compared to their non-flavonoid counterparts. Epiafzelechin and epicatechin were considered safer for oral consumption, while lupeol and stigmasterol were categorized as harmful if swallowed [45]. To the best of our knowledge, apart from one study [46] where epiafzelechin was among the metabolites docked against the PPARɣ receptor (PDB ID 2Q5S), no other study is available on the molecular docking of epiafzelechin against antidiabetic receptors. This study explored epiafzelechin’s potential in molecular docking against diabetes-related protein receptors. Epiafzelechin is a type B oligomer propelargonidin [47], and pelargonidin and its glycosides have been demonstrated to possess antidiabetic potential by reducing hyperglycemia and glycation levels as well as stimulating insulin secretion in rodent pancreatic β-cells in vitro [48,49,50], inferring the possible bioactivity of epiafzelechin. The molecular docking study could not unravel the potential activity of epiafzelechin in the same manner it failed with conventional therapeutics, indicating its limitations. However, this study suggests that epiafzelechin alone or in synergy with other compounds could be considered a potential drug candidate for diabetes treatment.

## 4. Materials and Methods

All the reagents used in this study are ACS grade and purchased from Sigma, Johannesburg, South Africa unless otherwise stated.

### 4.1. General

Nuclear Magnetic Resonance (NMR) spectra, both ^1^H and ^13^C, were recorded using a Bruker (Billerica, MA, USA) spectrometer at 500 MHz and a Variant spectrometer at 400 MHz. Chemical shifts (δ) are referenced in parts per million (ppm) against the internal standard tetramethylsilane (TMS). Column chromatography utilized MN silica gel 60 (0.063–0.2 mm/70–230 mesh), and preparative thin layer chromatography (TLC) was conducted with high-purity grade powder silica gel (60 A, 2–25 µm) from Sigma-Aldrich, Darmstadt, Germany. TLC plates of silica gel 60 F254 (Merck, Darmstadt, Germany) were employed for monitoring fractions. The detection of spots was accomplished using UV light (254 and 365 nm), followed by spraying with 30% H_2_SO_4_ and heating up to 110 °C.

### 4.2. Extraction and Isolation

The ethyl acetate fraction, obtained through solvent-solvent fractionation and reduced in a vacuum rotary evaporator to a semi-dried mass, yielded 15 g of dried extract. This extract was subjected to silica gel column chromatography and eluted with increasing polarities of n-hexane (n-hex), ethyl acetate (EtOAc), and methanol. This process resulted in 115 fractions of 500 mL each. Fraction 18 eluted with n-hex: EtOAc (85:15) crystallized in the same solvent system to yield 1 (23 mg), while a combined fraction 16–19 [n-hex: EtOAc (90:10, 85:15)] and 20–25 [n-hex: EtOAc (80:20)] crystallized also in n-hex: EtOAc (85:15) to afford 1 (10 mg) and 2 (25 mg), respectively. Fractions 10–30 (1 g) eluted with n-hex: EtOAc (95:5) were further subjected to purification silica gel column chromatography using n-hexane and EtOAc (0–100%) to afford 139 fractions of 50 mL each, and sub-fraction 76 (23 mg) yielded 3 (17.4 mg) after preparative TLC. Fractions 46–52 eluted with n-hex: EtOAc (70:30) were also subjected to similar silica gel column chromatography as fractions 10–30, followed by preparative TLC to afford 4 (15 mg) and 5 (11 mg).

### 4.3. Preparation of Samples

A solution of each isolated compound was separately made with dimethyl sulfoxide to produce a 100 mg/mL stock solution and dissolved appropriately to produce working solutions for the following assays. 

### 4.4. Phytochemical Screening

Each of the isolated compounds was individually dissolved in methanol (1 mg/mL) and utilized for the preliminary phytochemical screening to identify the class according to the previously described method [51]. The compounds were assessed for steroids and terpenoids by Liebermann-Burchard’s test. To 1 mL of the compound solution were added chloroform (1 mL), acetic anhydride (2 mL), and concentrated sulfuric acid (2 drops). A change of color to dark green signifies the presence of steroids, while a change to dark pink or red signifies the presence of terpenes. The compounds were assessed for flavonoids by Shinoda’s test. To 2 mL of compound solution, a piece of magnesium ribbon and 1 mL of concentrated hydrochloric acid were added. A pinkish-red or red color of the solution indicates the presence of flavonoids.

### 4.5. α-Glucosidase Inhibition Assay

The effect of the isolated compounds on the activity of the α-glucosidase enzyme was evaluated by the method of Olaokun et al. [13]. Firstly, a solution of 2.5 mg of compound in 1 mL of 50% dimethyl sulfoxide was made. A compound solution (100 μL) and sucrose (200 μL of a 56 mM solution) in 0.1 M PO4 buffer (pH 7) were incubated at 37 °C for 5 min. Then 200 μL of rat intestinal α-glucosidase solution was added with vigorous shaking before incubation for 20 min. Thereafter, 750 μL of 2 M Tris-HCl buffer (pH 6.9) was added to the mixture. Positive control (acarbose, 1 mg/mL), sample blank, and solvent control were included. The quantity of glucose released was estimated using the glucose oxidase method, with absorbance recorded at 540 nm. The percentage of α-glucosidase activity was calculated from Equation (1) and thereafter, the concentration of the compound that inhibited 50% of the enzyme activity (IC_50_).

(1) $$\%Inhibition=100\times \left(\frac{{∆A}_{Control}-{∆A}_{Sample}}{{∆A}_{Control}}\right)\;{∆A}_{Control}=A_{Test}-A_{Blank}\;{∆A}_{Sample}=A_{Test}-A_{Blank}$$

### 4.6. Glucose Utilization Activity

The potential of compounds to stimulate cells to utilize glucose was estimated using the method adopted by Olaokun et al. [15]. C2C12 muscle cells (2.5 × 10^−4^ cells/mL) and H-4-11-E liver cells (3.0 × 10^−4^ cells/mL) separately in Dulbecco’s modified eagle medium (Sigma) supplemented with 0.25% bovine serum albumin (Sigma) were dispensed (200 μL) into the 96-well plates. The C2C12 cells were incubated for 4 days, while the H-4-11-E cells were incubated for 2 days at 37 °C in a 5% CO_2_ incubator. Thereafter, the cells were examined and utilized for the glucose uptake assay. The compound solution (100 mg/mL) freshly dissolved in DMSO was further serially diluted (15–250 μg/mL) with growth medium prior to the assay. The treated cells were incubated for 1 h (C2C12) and 3 h (H-4-11-E). The positive control was insulin (Sigma) (1 μM) for C2C12 cells and metformin (Sigma) (1 µM) for H-4-11-E cells. Thereafter, the amount of glucose in the medium was evaluated using the glucose oxidase method, with absorbance recorded at 540 nm. The percentage of glucose utilized was calculated as the percentage change in absorbance in comparison to the untreated cells using Equation (2).

(2) $$\%Glucose\;utilized=100\times \left(\frac{{∆A}_{Control\;(untreated\;cells)}-{∆A}_{Sample\;(treated\;cells)}}{{∆A}_{Control\;(untreated\;cells)}}\right)$$

### 4.7. Statistical Analysis

The α-glucosidase inhibitory and glucose utilization assays were conducted in triplicate and repeated three times. Data are presented as the mean ± standard error of the mean, with statistical analysis carried out by one-way analysis of variance (ANOVA) and post hoc analysis by the student’s *t*-test. A significant difference was recorded with a value of *p* ≤ 0.05. 

### 4.8. In Silico Analyses of the Isolated Compounds

#### 4.8.1. Ligand and Target Protein Preparation, and Molecular Docking

The in-silico antidiabetic activity was conducted via molecular docking technique using AutoDock Vina between ligands and targeted enzymes involved in glucose metabolism to obtain free binding energy. 

The two-dimensional structure of the five isolated compounds and positive controls (acarbose, sitagliptin, rosiglitazone, gliclazide, and metformin) was retrieved from the website (https://pubchem.ncbi.nlm.nih.gov) (accessed 23 June 2022). These were then converted to three-dimensional forms with polar hydrogens and charges at physiological pH 7.4 added, followed by energy minimization and optimization with an MMFF94 force field using Chem 3D 15.0 PerkinElmer, 2011. Furthermore, Gasteiger charges were added to the three-dimensional structure of ligands and converted into pdbqt file format using the AutoDock Tools (The Scripps Research Institute, La Jolla, CA, USA) [52,53,54]. 

The 3-D of the various receptors: α-amylase (PDB: 1OSE), α-glucosidase (PDB: 2QMJ), glycogen phosphorylase (GP) (PDB: 1NOI); Peroxisome Proliferator-Activated Receptor Gamma (PPARɣ) (PDB: 3G9E), Dipeptidyl peptidase 4 (DPP-IV) (PDB: 2P8S), Glucokinase (GK) (PDB: 4RCH), Protein Tyrosine Phosphatase 1B (PTP1B) (PDB: 5T19), and glucose transporter 1 catalytic site (GLUT1) (PDB: 5EQG) were retrieved from the protein data bank. To specify docking regions, the coordinates of the grid boxes were set according to the binding sites of the co-crystallized ligand positions. However, for some proteins that lack co-crystallized ligands, the grid box was defined using DogSiteScorer by Proteins Plus Zentrum für Bioinformatik: Universität Hamburg (https://proteins.plus/) (accessed 23 June 2022) to identify potential binding sites. The cavity with the highest D-score value suggests more druggable sites that were selected as docking regions. The docking study was validated by re-docking the reference ligand into the appropriate protein cavity, and acceptable where the root mean square deviation (RMSD) value is <2.0 Å [25]. Analysis and visualization of the interaction were performed using PyMOL version: 2.4 (https://pymol.org/2/) (accessed 23 June 2022). After docking, the ligand-receptor conformations selected for visualization were those with the lowest free binding energy (ΔG kcal/mol), noting the number of hydrogen bonding, type interactions, and total interactions. The analysis of the ligand-receptor conformations for their molecular interactions was conducted in two dimensions.

#### 4.8.2. ADMET Profiling of the Isolated Compounds

The simplified molecular-input line-entry system (SMILES) formats of each isolated compound were retrieved from the PubChem server (https://pubchem.ncbi.nlm.nih.gov/) (accessed on 23 July 2022) and used for in silico prediction. The physicochemical and pharmacokinetic properties, i.e., ADME profiles (absorption, distribution, metabolism, and excretion), were run on the SwissADME web server (http://www.swissadme.ch/index.php) [19] (accessed on 23 June 2022) and the pkCSM web server (https://biosig.lab.uq.edu.au/pkcsm/) [31] (accessed on 23 July 2022). The drug-likeness of compounds based on Lipinski’s rule of five was also predicted with the SwissADME web server, while the toxicity analysis was carried out by the ProTox-II web server (https://tox-new.charite.de/protox_II/) [55] (accessed on 23 July 2022).

## 5. Conclusions

The present study highlights the antidiabetic potential of compounds isolated from *F. lutea’s* ethyl acetate fraction. Their inhibitory effects on α-glucosidase activity, glucose utilization enhancement, and apparent binding interactions with key receptors emphasize their therapeutic promise. While epiafzelechin and epicatechin exhibit optimal drug-like properties, further studies focusing on harnessing their synergistic effects with other compounds are necessary. Also, while the findings of this study showed potential for compounds like epiafzelechin and epicatechin in the ethyl acetate fraction of *F. lutea* as candidates for antidiabetic agents, it is important to note that the scope of this study is limited. Further comprehensive investigations, including additional in-depth studies, are essential to substantiate and validate the efficacy and safety of these molecules as potential therapeutic options for diabetes.

## Figures and Tables

**Table 1:** Qualitative phytochemical screening of isolated compounds.

Compounds	Terpenoids	Steroids	Flavonoids
Compound **1**	+	−	−
Compound **2**	−	+	−
Compound **3**	+	−	−
Compound **4**	−	−	+
Compound **5**	−	−	+

+ = present, − = absent.

**Table 2:** α-Glucosidase inhibitory activity of isolated compounds.

Compound	IC_50_ (µg/mL)
Lupeol	>1000
Stigmasterol	115.71 ± 11.6 ^a^
α-Amyrin acetate	335.82 ± 22.6 ^a^
Epicatechin	5.72 ± 2.6 ^b^
Epiafzelechin	7.64 ± 4.9 ^b^

^a,b^ No significant difference between extracts with the same value, but significant difference *p* < 0.05 between different values. IC_50_ for acarbose positive control = 1.52 ± 0.05 µg/mL.

**Table 3:** Free energy of binding interaction of target receptors with isolated compounds.

Compounds	Free Energy of Binding ΔG (kcal/mol) with Target Receptors							
	1NOI	3G9E	2P8S	5EQG	4RCH	5T19	1OSE	2QMJ
Lupeol	−9.3	−8.1	−9.5	−10.8	−8.1	−8.3	−10	−7.8
Stigmasterol	−8.3	−8.6	−9.0	−11.2	−9.2	−7.8	−10.6	−9.1
a-Amyrin-acetate	−8.5	−7.9	−9.4	−10.1	−7.7	−7.6	−9.2	−7.5
Epicatechin	−8.1	−7.6	−7.8	−8.6	−8.0	−7.5	−8.9	−7.2
Epiafzelechin	−8.2	−7.6	−7.6	−8.7	−8.6	−7.7	−8.8	−7.0
Native ligand	−7.2	−9.4	−9.4	−9.9	−8.5	−9.5	−9.8	−7.5

Glycogen phosphorylase (1NOI); Peroxisome Proliferator-Activated Receptor Gamma (3G9E), Dipeptidyl peptidase 4 (DPP-IV) (2P8S), Protein Tyrosine Phosphatase 1B (5T19), GLUT1 catalytic site (5EQG), Glucokinase (4RCH), α-Amylase (1OSE), and α-Glucosidase (2QMJ). Free energy of binding (ΔG kcal/mol) for positive controls—acarbose (1OSE,−8.0; 2QMJ, −7.1), sitagliptin (2P8S, −8.2), Rosiglitazone (3G9E, −8.8), and Metformin (1NOI, −5.0).

**Table 4:** In silico physicochemical and drug-likeness properties of isolated compounds.

Parameters	Compounds				
	Epiafzelechin	Epicatechin	Lupeol	Stigmasterol	α-Amyrin-Acetate
Molecular weight (MW) (g/mol)	274.3	290.3	426.7	412.7	468.8
Fraction Csp^3^	0.2	0.2	0.93	0.86	0.91
#Rotatable bonds	1	1	1	5	2
#H-bond acceptors	5	6	1	1	2
#H-bond donors	4	5	1	1	0
Molecular refractivity	72.31	74.33	135.14	132.76	144.88
Topological Polar Surface Area (Å^2^)	90.15	110.38	20.23	20.23	26.3
Lipophilicity Log P_o/w_	1.84	1.55	8.02	7.8	8.6
Water solubility Log S (Ali)	Soluble	Soluble	Insoluble	Poorly soluble	Insoluble
Drug likeness (Lipinski rule), #violations	Yes, 0	Yes, 0	Yes, 1	Yes, 1	Yes, 1
Bioavailability Score	0.55	0.55	0.55	0.55	0.55
Leadlikeness #violations	0	0	2	2	2

Csp^3^ = Fraction of carbon atoms in the sp^3^ hybridisation, H = Hydrogen, # = number, lead likeness violation: MW > 350, log P > 5.

**Table 5:** ADME pharmacokinetic properties of the isolated compounds.

Parameters	Compounds				
	Epiafzelechin	Epicatechin	Lupeol	Stigmasterol	α-Amyrin-Acetate
Absorption					
Water solubility (log mol/L)	−3.254	−3.117	−5.861	−6.682	−6.67
Caco2 permeability (log Papp in 10^−6^ cm/s)	1.077	−0.283	1.226	1.213	1.222
GI absorption	High	High	Low	Low	Low
Skin Permeability log Kp (cm/s)	−2.735	−2.735	−2.744	−2.783	−2.82
P-gp substrate (Yes/No)	Yes	Yes	No	No	No
P-gp I inhibitor (Yes/No)	No	No	Yes	Yes	Yes
P-gp II inhibitor (Yes/No)	No	No	Yes	Yes	Yes
Distribution					
VDss (human) (log L/kg)	0.562	1.027	0	0.178	0.148
Fraction unbound (human)	0.194	0.235	0	0	0
BBB permeant (log BB)	−0.818	−1.054	0.726	0.771	0.599
CNS permeability (log PS)	−2.473	−3.298	−1.714	−1.652	−1.963
Metabolism					
CYP2D6 substrate	No	No	No	No	No
CYP3A4 substrate	No	No	Yes	Yes	Yes
CYP1A2 inhibitor	No	No	No	No	No
CYP2C19 inhibitor	No	No	No	No	No
CYP2C9 inhibitor	No	No	No	No	No
CYP2D6 inhibitor	No	No	No	No	No
CYP3A4 inhibitor	No	No	No	No	No
Excretion					
Total renal clearance (log mL/min/kg)	0.255	0.183	0.153	0.618	0.025
Renal OCT2 substrate	No	No	No	No	No

OCT2 = Organic Cation Transporter 2; BBB = Blood-brain barrier, CNS = Central nervous system, P-gp = P-glycoprotein, VDss = steady-state volume of distribution.

**Table 6:** Toxicity properties of the isolated compounds.

Parameters	Compounds				
	Epiafzelechin	Epicatechin	Lupeol	Stigmasterol	α-Amyrin-Acetate
Max. tolerated dose (human) (log mg/kg/day)	0.136	0.438	−0.502	−0.664	−0.485
hERG I inhibitor	No	No	No	No	No
hERG II inhibitor	No	No	Yes	Yes	Yes
Oral Rat Acute Toxicity (LD_50_) (mol/kg)	2.365	2.428	2.563	2.54	2.25
Oral Rat Chronic Toxicity (LOAEL) (log mg/kg bw/day)	2.215	2.5	0.89	0.872	2.039
AMES toxicity	No	No	No	No	No
Hepatotoxicity	No	No	No	No	No
Skin Sensitization	No	No	No	No	No
Immunotoxicity	No	No	Yes	Yes	Yes
*T. pyriformis* toxicity (log µg/L)	0.519	0.347	0.316	0.433	0.359
Minnow toxicity (log mM)	2.75	3.585	−1.696	−1.675	−1.996
Predicted LD_50_ (mg/kg)	2500	10,000	2000	890	3460
Predicted Toxicity Class	5	6	4	4	5

hERG = human ether-go-go-related gene.

## Data Availability

The data in this study are available in the article.

## Supplementary Materials

The following supporting information can be downloaded at: https://www.mdpi.com/article/10.3390/molecules28237717/s1.

## References

[1] Mutyambizi C., Booysen F., Stokes A., Pavlova M., Groot W. (2019). Lifestyle and Socio-Economic Inequalities in Diabetes Prevalence in South Africa: A Decomposition Analysis. PLoS ONE.

[2] World Health Organization (2011). Global Status Report on Noncommunicable Diseases 2010.

[3] World Health Organization (1999). Consultation, Definition, diagnosis and classification of diabetes mellitus and its complications. Part 1: Diagnosis and Classification of Diabetes Mellitus.

[4] Amos A.F., McCarty D.J., Zimmet P. (1997). The Rising Global Burden of Diabetes and Its Complications: Estimates and Projections to the Year 2010. Diabet. Med..

[5] Statistics South Africa (2015). Mortality and Causes of Death in South Africa, Findings from Death Notification.

[6] (2017). IDF Diabetes Atlas, 8th ed. http://www.diabetesatlas.org/resources/2017-atlas.html.

[7] Odeyemi S., Bradley G. (2018). Medicinal Plants Used for the Traditional Management of Diabetes in the Eastern Cape, South Africa: Pharmacology and Toxicology. Molecules.

[8] Olaokun O.O. (2012). The Value of Extracts of *Ficus lutea* (Moraceae) in the Management of Type II Diabetes in a Mouse Obesity Model. Ph.D. Dissertation.

[9] Tabasum S., Khare S. (2016). Safety of medicinal plants: An important concern. Int. J. Pharma Bio. Sci..

[10] Nyakudya T.T., Tshabalala T., Dangarembizi R., Erlwanger K.H., Ndhlala A.R. (2020). The Potential Therapeutic Value of Medicinal Plants in the Management of Metabolic Disorders. Molecules.

[11] Watt J.M., Breyer-Brandwijk M.G. (1962). Medicinal and Poisonous Plants of Southern and Eastern Africa.

[12] Ramadan M., Ahmad A.S., Nafady A.M., Mansour A. (2009). Chemical Composition of the Stem Bark and Leaves of *Ficus Pandurata* Hance. Nat. Prod. Res..

[13] Olaokun O.O., McGaw L.J., Eloff J.N., Naidoo V. (2013). Evaluation of the Inhibition of Carbohydrate Hydrolysing Enzymes, Antioxidant Activity and Polyphenolic Content of Extracts of Ten African *Ficus* Species (Moraceae) Used Traditionally to Treat Diabetes. BMC Complement. Altern. Med..

[14] Coates-Palgrave K. (2002). Moraceae (The fig and mulberry family). Keith Coates-Palgrave Trees of Southern Africa Cape Town: 2nd imp.

[15] Olaokun O.O., McGaw L.J., Awouafack M.D., Eloff J.N., Naidoo V. (2014). The Potential Role of GLUT4 Transporters and Insulin Receptors in the Hypoglycaemic Activity of *Ficus Lutea* Acetone Leaf Extract. BMC Complement. Altern. Med..

[16] Olaokun O.O., McGaw L.J., Janse van Rensburg I., Eloff J.N., Naidoo V. (2016). Antidiabetic Activity of the Ethyl Acetate Fraction of *Ficus lutea* (Moraceae) Leaf Extract: Comparison of an in Vitro Assay with an In Vivo Obese Mouse Model. BMC Complement. Altern. Med..

[17] Mahato S.B., Kundu A.P. (1994). ^13^C NMR Spectra of Pentacyclic Triterpenoids—A Compilation and Some Salient Features. Phytochemistry.

[18] Poumale H.M.P., Songfack Djoumessi A.V.B., Ngameni B., Sandjo L.P., Ngadjui B.T., Shiono Y. (2011). A New Ceramide Isolated from *Ficus lutea* Vahl (moraceae). Acta Chim. Slov..

[19] Forgo P., Kövér K.E. (2004). Gradient enhanced selective experiments in the 1H NMR chemical shift assignment of the skeleton and side-chain resonances of stigmasterol, a phytosterol derivative. Steroids.

[20] Zhao M., Yang B., Wang J., Li B., Jiang Y. (2006). Identification of the Major Flavonoids from Pericarp Tissues of Lychee Fruit in Relation to Their Antioxidant Activities. Food Chem..

[21] Du X., Li Y., Xia Y.-L., Ai S.-M., Liang J., Sang P., Ji X.-L., Liu S.-Q. (2016). Insights into Protein–Ligand Interactions: Mechanisms, Models, and Methods. Int. J. Mol. Sci..

[22] Lipinski C.A., Lombardo F., Dominy B.W., Feeney P.J. (2012). Experimental and Computational Approaches to Estimate Solubility and Permeability in Drug Discovery and Development Settings. Adv. Drug. Deliv. Rev..

[23] Vo Van L., Pham E.C., Nguyen C.V., Duong N.T.N., Vi Le Thi T., Truong T.N. (2022). In Vitro and In Vivo Antidiabetic Activity, Isolation of Flavonoids, and in Silico Molecular Docking of Stem Extract of *Merremia Tridentata* (L.). Biomed. Pharmacother..

[24] Daina A., Michielin O., Zoete V. (2017). SwissADME: A Free Web Tool to Evaluate Pharmacokinetics, Drug-Likeness and Medicinal Chemistry Friendliness of Small Molecules. Sci. Rep..

[25] Athar M., Sona A.N., Bekono B.D., Ntie-Kang F. (2019). Fundamental Physical and Chemical Concepts behind “Drug-Likeness” and “Natural Product-Likeness”. Phys. Sci. Rev..

[26] Johnson D.E., Wolfgang G.H.I. (2000). Predicting Human Safety: Screening and Computational Approaches. Drug. Discov. Today.

[27] Pathak M., Ojha H., Tiwari A.K., Sharma D., Saini M., Kakkar R. (2017). Design, Synthesis and Biological Evaluation of Antimalarial Activity of New Derivatives of 2,4,6-s-Triazine. Chem. Cent. J..

[28] Naspiah N., Rizki Fadhil Pratama M., Sukardiman S.S. (2021). Xanthine Oxidase Inhibition Activity and ADMET Properties of Terap (Artocarpus Odoratissimus Blanco) Leaves Metabolites: Phytochemical Screening and in Silico Studies. Pharmacogn. J..

[29] Martin Y.C. (2005). A Bioavailability Score. J. Med. Chem..

[30] Ngbolua J.P.K.T.N., Kilembe J.T., Matondo A., Ashande C.M., Mukiza J., Nzanzu C.M., Ruphin F.P., Baholy R., Mpiana P.T., Mudogo V. (2022). In Silico Studies on the Interaction of Four Cytotoxic Compounds with Angiogenesis Target Protein HIF-1α and Human Androgen Receptor and Their ADMET Properties. Bull. Natl. Res. Cent..

[31] Brenk R., Schipani A., James D., Krasowski A., Gilbert I., Frearson J., Wyatt P. (2008). Lessons Learnt from Assembling Screening Libraries for Drug Discovery for Neglected Diseases. ChemMedChem.

[32] Schneider G. (2002). Trends in Virtual Combinatorial Library Design. Curr. Med. Chem..

[33] Bucao X.E., Solidum J. (2021). In Silico Evaluation of Antidiabetic Activity and ADMET Prediction of Compounds from *Musa Acuminata* Colla Peel. Philipp. J. Sci..

[34] Ottaviani G., Gosling D.J., Patissier C., Rodde S., Zhou L., Faller B. (2010). What Is Modulating Solubility in Simulated Intestinal Fluids?. Euro. J. Pharm. Sci..

[35] Artursson P., Neuhoff S., Matsson P., Tavelin S., Taylor J.B., Triggle D.J. (2007). Passive permeability and active transport models for the prediction of oral absorption. Comprehensive Medicinal Chemistry II.

[36] Pires D.E.V., Blundell T.L., Ascher D.B. (2015). PkCSM: Predicting Small-Molecule Pharmacokinetic and Toxicity Properties Using Graph-Based Signatures. J. Med. Chem..

[37] Yeni Y., Rachmania R.A. (2022). The Prediction of Pharmacokinetic Properties of Compounds in *Hemigraphis Alternata* (Burm.F.) T. Ander Leaves Using PkCSM. Indones. J. Chem..

[38] Ghannay S., Kadri A., Aouadi K. (2020). Synthesis, in Vitro Antimicrobial Assessment, and Computational Investigation of Pharmacokinetic and Bioactivity Properties of Novel Trifluoromethylated Compounds Using in Silico ADME and Toxicity Prediction Tools. Monatsh. Chem.-Chem. Mon..

[39] Awortwe C., Fasinu P.S., Rosenkranz B. (2014). Application of Caco-2 Cell Line in Herb-Drug Interaction Studies: Current Approaches and Challenges. J. Pharm. Pharm. Sci..

[40] Heuberger J., Schmidt S., Derendorf H. (2013). When Is Protein Binding Important?. J. Pharm. Sci..

[41] Bhosle V.K., Altit G., Autmizguine J., Chemtob S., Polin R.A., Abman S.H., Rowitch D.H., Benitz W.E., Fox W.W. (2017). “Basic Pharmacologic Principles” in Fetal and Neonatal Physiology.

[42] Sadeghi M., Moradi M., Madanchi H., Johari B. (2021). In Silico Study of Garlic (*Allium sativum* L.)-Derived Compounds Molecular Interactions with α-Glucosidase. Silico Pharmacol..

[43] [OECD] Organisation for Economic Co-operation and Development (2018). Test no. 452: Chronic toxicity studies. OECD Guidelines for the Testing of Chemicals, Section 4.

[44] Ferrari I.V., Di Mario M. (2022). Prediction of physicochemical property/Biological Activity and ADMET (absorption, distribution, mechanism, excretion, and toxicity) parameters of approved HIV Medications. Int. J. Sci. Res. Biol. Sci..

[45] Wadanambi P.M., Seneviratne K.N., Jayathilaka N. (2023). In Silico Evaluation of Coconut Milk Phenolic Antioxidants and Their Inhibition of Oxidative Stress in *Intestinal lactobacillus* spp. In Vitro Chem. Pap..

[46] Rashied R.M.H., Abdelfattah M.A.O., El-Beshbishy H.A., El-Shazly A.M., Mahmoud M.F., Sobeh M. (2022). Syzygium Samarangense Leaf Extract Exhibits Distinct Antidiabetic Activities: Evidences from in Silico and in Vivo Studies. Arab. J. Chem..

[47] Das G., Nath R., Talukdar A.D., Ağagündüz D., Yılmaz B., Capasso R., Shin H., Patra J.K. (2023). Major Bioactive Compounds from Java Plum Seeds: An Investigation of Its Extraction Procedures and Clinical Effects. Plants.

[48] Roy M., Sen S., Chakraborti A.S. (2008). Action of Pelargonidin on Hyperglycemia and Oxidative Damage in Diabetic Rats: Implication for Glycation-Induced Hemoglobin Modification. Life Sci..

[49] Vinayagam R., Xu B. (2015). Antidiabetic Properties of Dietary Flavonoids: A Cellular Mechanism Review. Nutr. Metab..

[50] Jayaprakasam B., Vareed S.K., Olson L.K., Nair M.G. (2004). Insulin Secretion by Bioactive Anthocyanins and Anthocyanidins Present in Fruits. J. Agric. Food Chem..

[51] Kumar G.S., Jayaveera K.N., Kumar C.K., Sanjay U.P., Swamy B.M., Kumar D.V. (2007). Antimicrobial Effects of Indian Medicinal Plants against Acne-Inducing Bacteria. Trop. J. Pharm. Res..

[52] Olaokun O.O., Manonga S.A., Zubair M.S., Maulana S., Mkolo N.M. (2022). Molecular Docking and Molecular Dynamics Studies of Antidiabetic Phenolic Compound Isolated from Leaf Extract of *Englerophytum magalismontanum* (Sond.) T.D.Penn. Molecules.

[53] Morris G.M., Goodsell D.S., Halliday R.S., Huey R., Hart W.E., Belew R.K., Olson A.J. (1998). Automated Docking Using a Lamarckian Genetic Algorithm and an Empirical Binding Free Energy Function. J. Comput. Chem..

[54] Meneses L., Cuesta S. (2015). Determinación computacional de la afinidad y eficiencia de enlace de antinflamatorios no esteroideos inhibidores de la Ciclooxigenasa-2. Rev. Ecuat. Med. Y Cienc. BiolóGicas.

[55] Banerjee P., Eckert A.O., Schrey A.K., Preissner R. (2018). ProTox-II: A Webserver for the Prediction of Toxicity of Chemicals. Nucleic Acids Res..

