# Supplementary material for: Antidiabetic Activity, Molecular Docking, and ADMET Properties of Compounds Isolated from Bioactive Ethyl Acetate Fraction of Ficus lutea Leaf Extract
Source: Molecules. 2023 Nov 22;28(23):7717. doi: 10.3390/molecules28237717 (PMC10708125; doi:10.3390/molecules28237717)
Supplement: Supplementary file 1 [file molecules-28-07717-s001.zip › molecules-2682502-supplementary.pdf]

Supplementary data

NMR Spectra of compound isolated from *Ficus lutea*

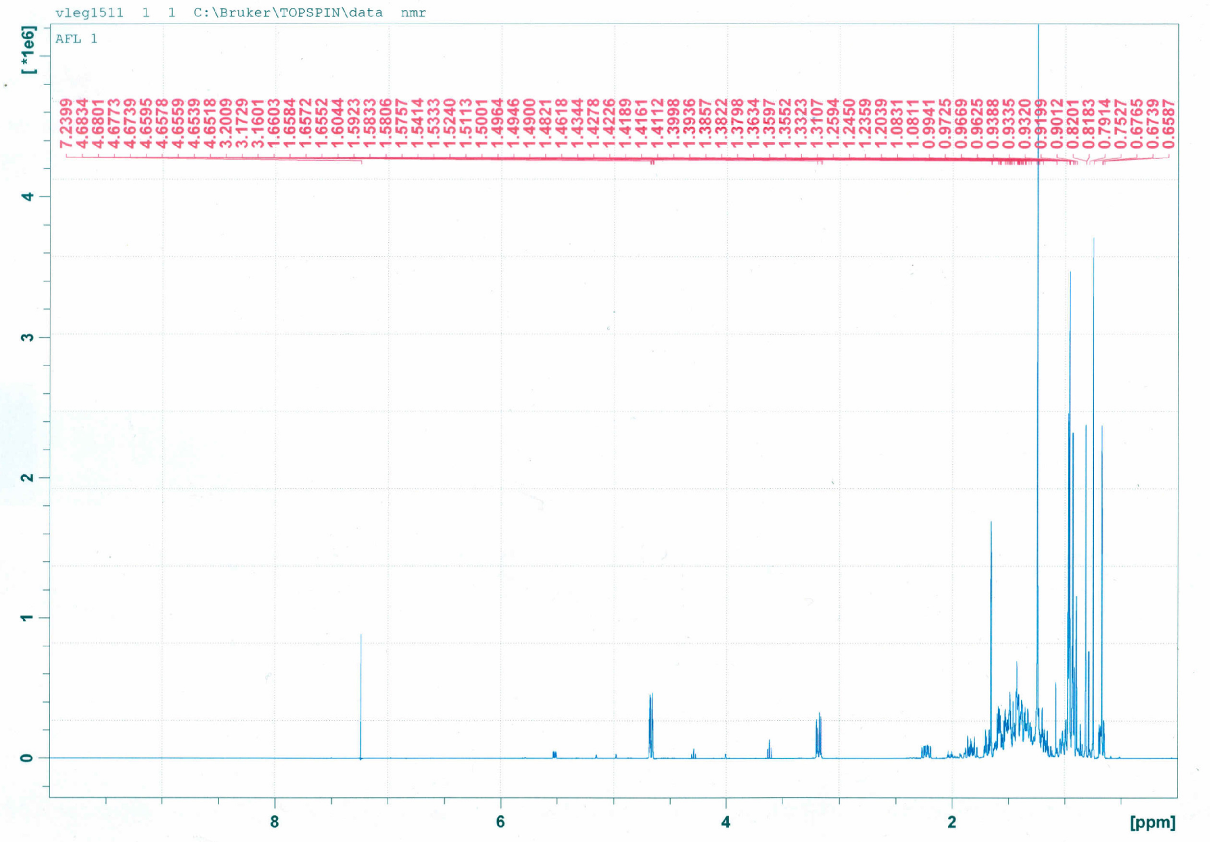

Figure S1: ^{1}H-NMR (500 MHz, $CDCl_3$) of Lupeol

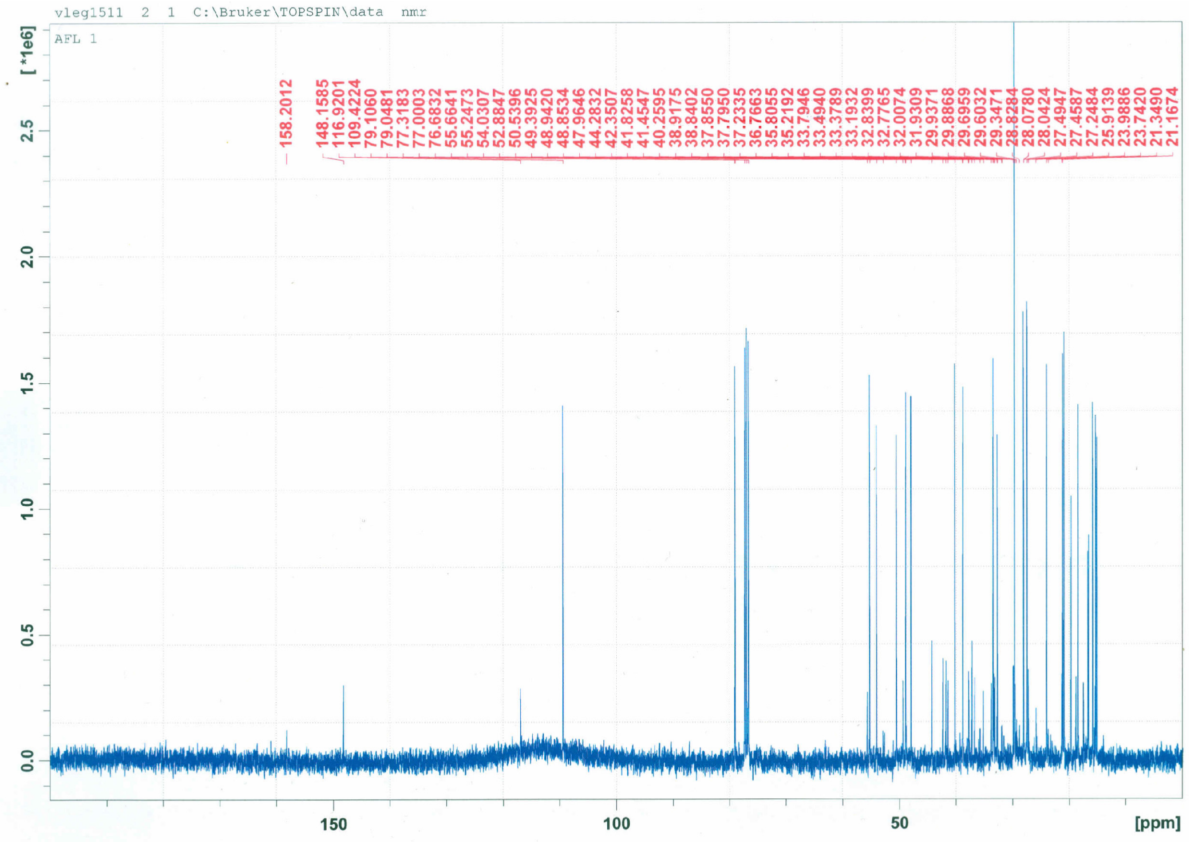

Figure S2: ^{13}C-NMR (125 MHz, $CDCl_3$) Spectrum of Lupeol

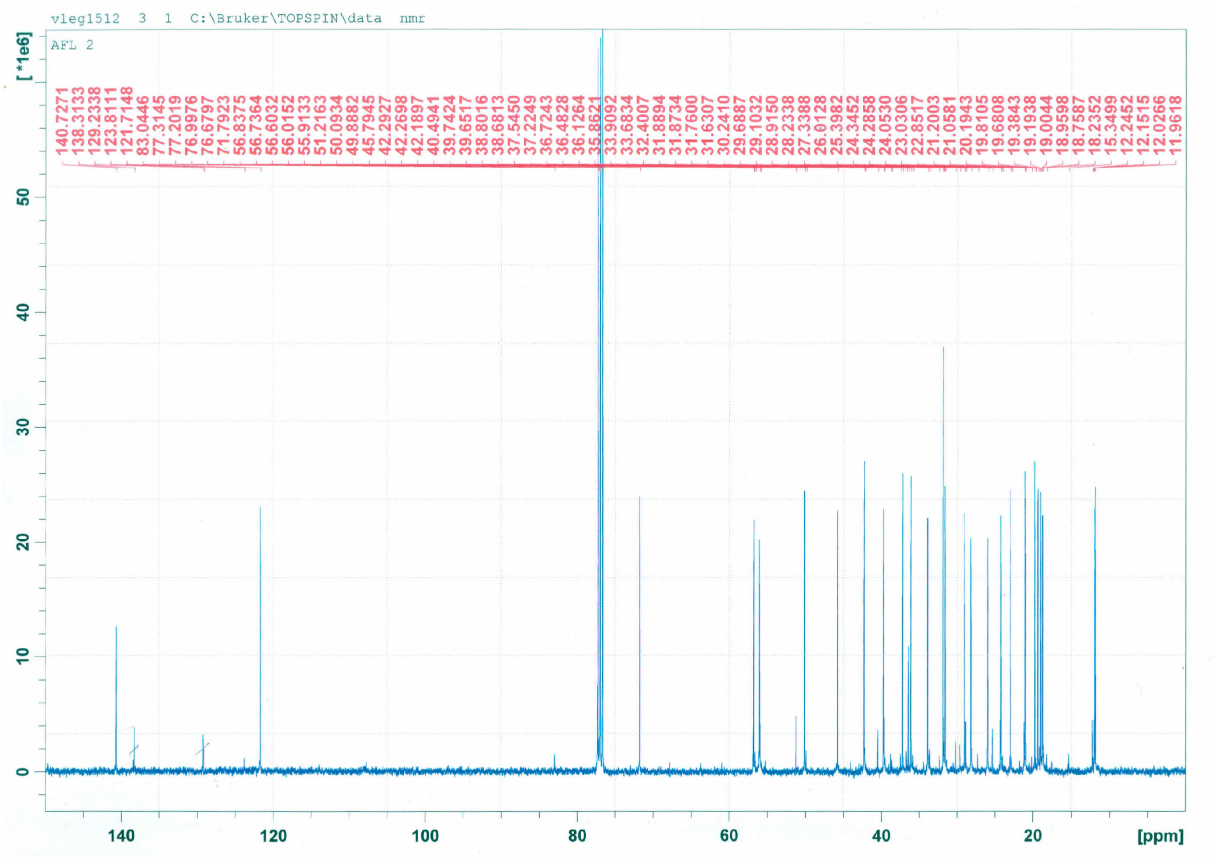

Figure S3: ^{13}C-NMR (125 MHz, $CDCl_3$) of stigmasterol

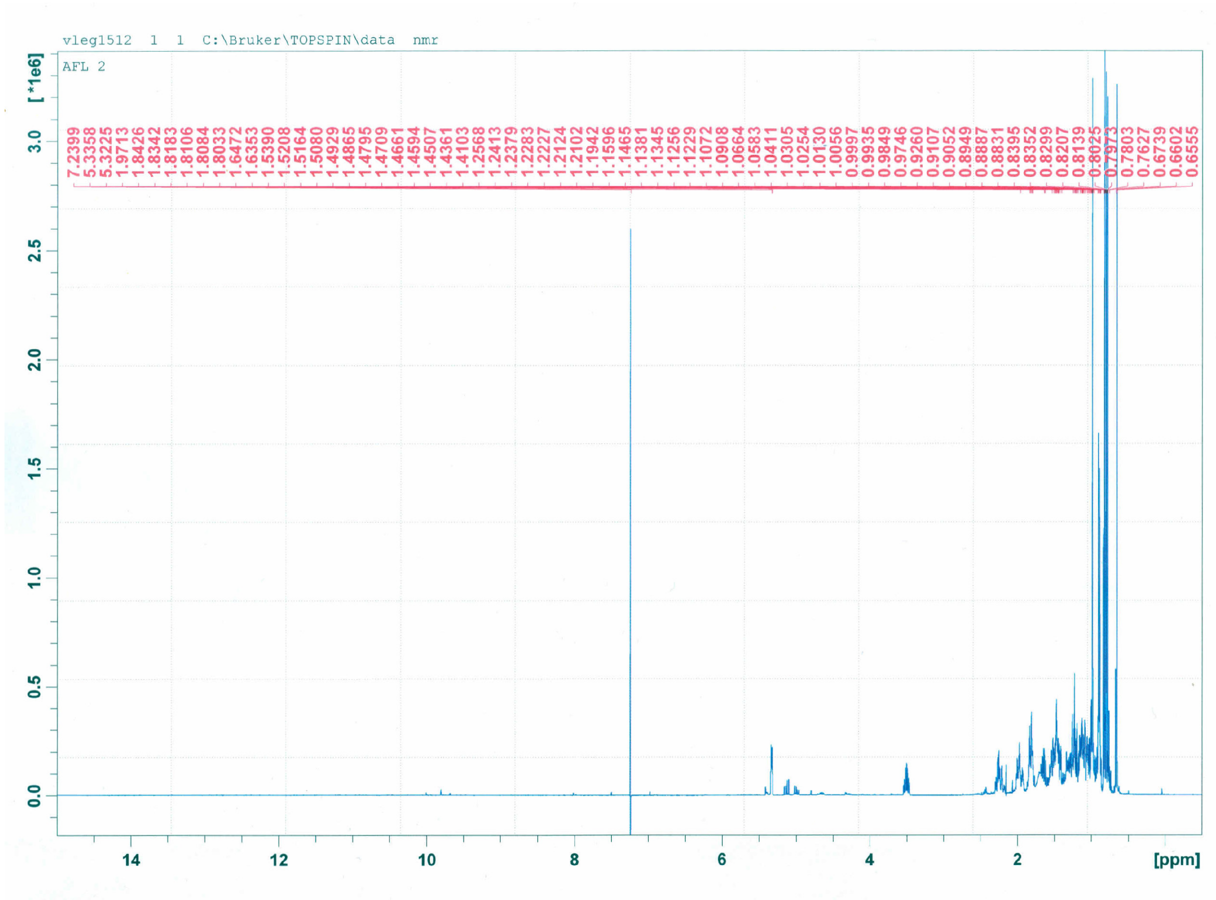

Figure S4: ^{1}H-NMR (500 MHz, $CDCl_3$) Spectrum of Stigmasterol

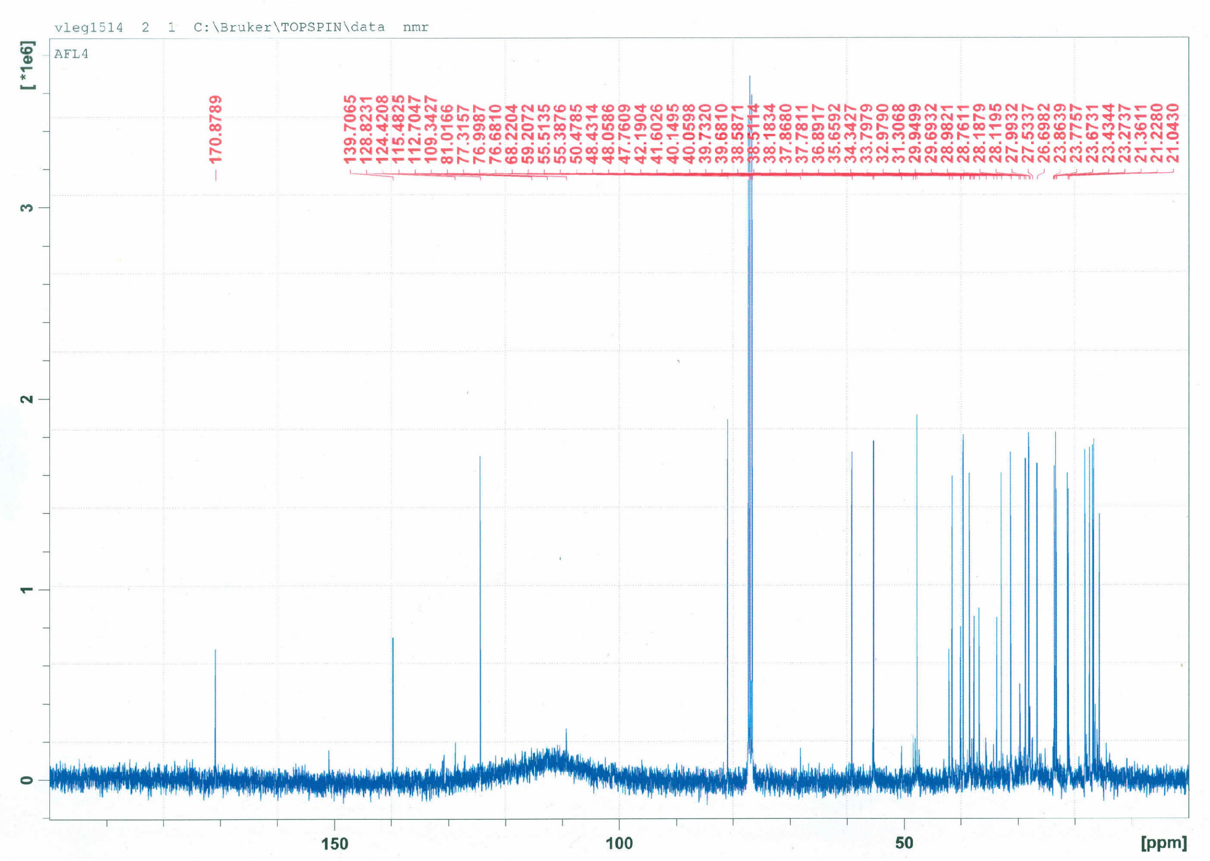

Figure S5: ^{13}C-NMR (125 MHz, $CDCl_3$) Spectrum of α-amyrin acetate

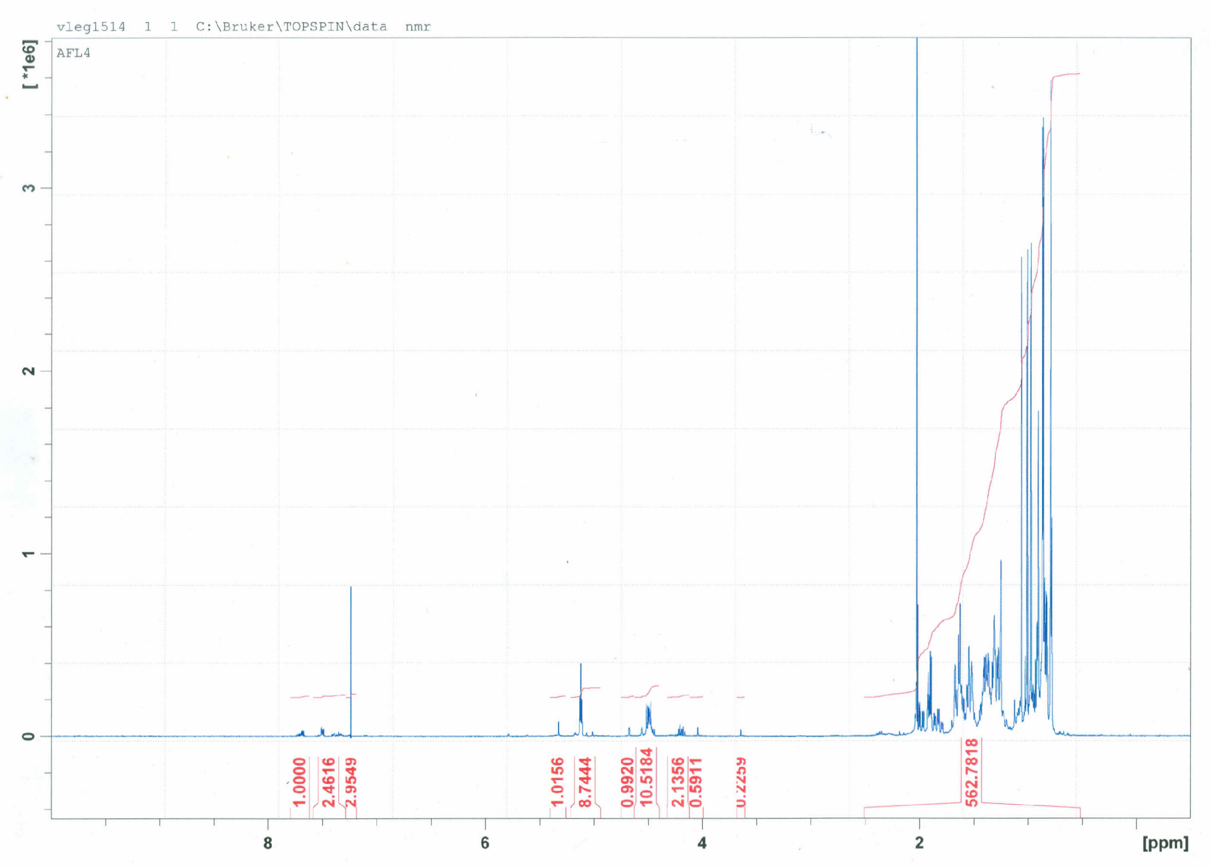

Figure S6: ^{1}H NMR (500 MHz, $CDCl_3$) Spectrum of α-amyrin acetate

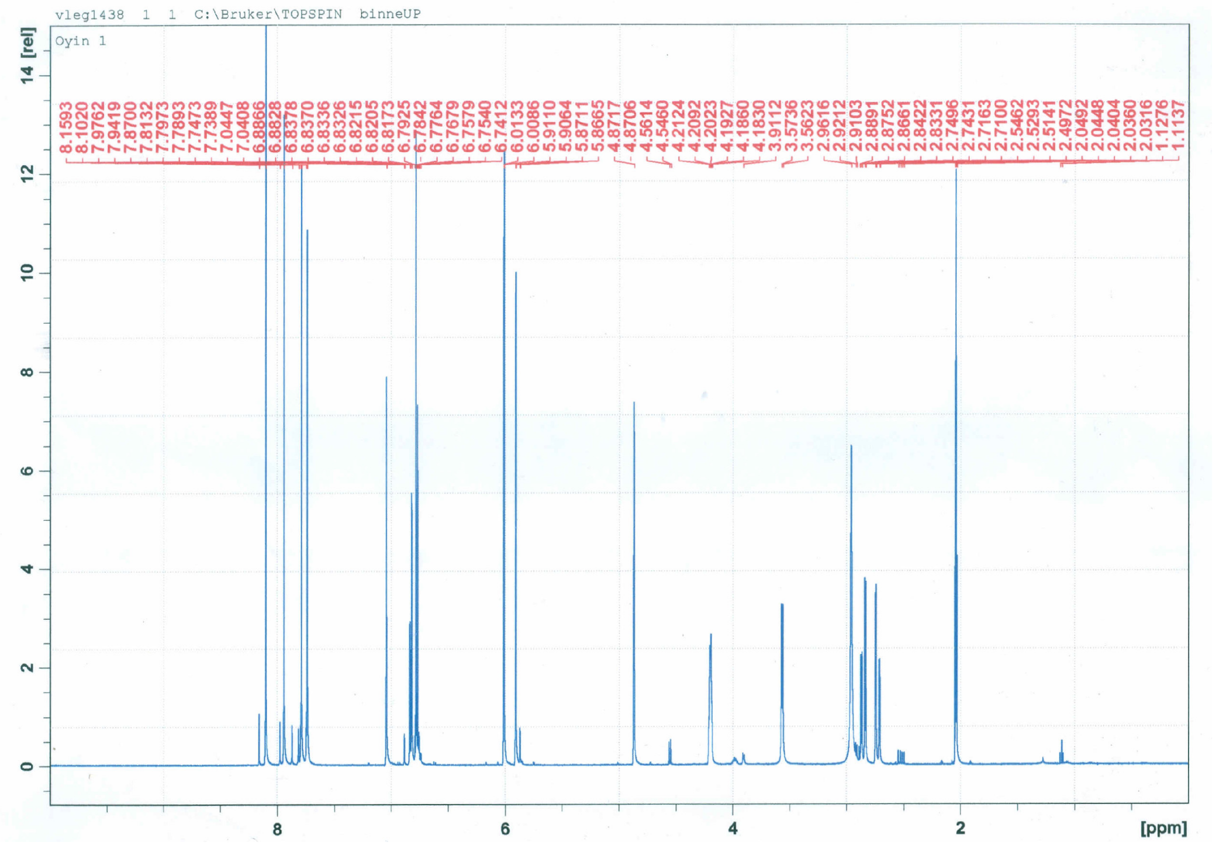

Figure S7: ^{1}H NMR (500 MHz, $CDCl_3$) Spectrum of Epicatechin

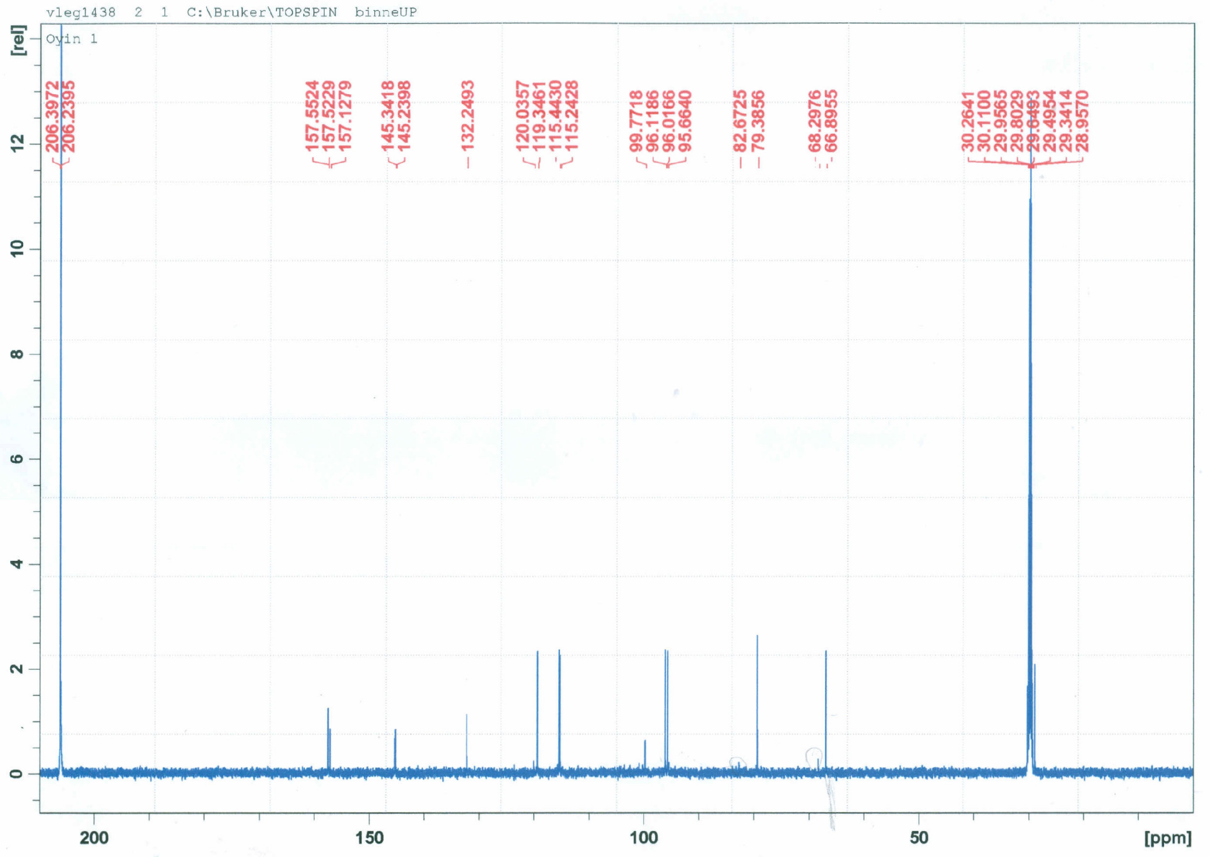

Figure S8: ^{13}C-NMR (125 MHz, $CDCl_3$) Spectrum of Epicatechin (**4**)
